# *Thymus algeriensis* and *Artemisia herba-alba* Essential Oils: Chemical Analysis, Antioxidant Potential and In Vivo Anti-Inflammatory, Analgesic Activities, and Acute Toxicity

**DOI:** 10.3390/molecules26226780

**Published:** 2021-11-10

**Authors:** Khadija El Ouahdani, Imane Es-safi, Hamza Mechchate, Mohammed Al-zahrani, Ashraf Ahmed Qurtam, Mohammed Aleissa, Amina Bari, Dalila Bousta

**Affiliations:** 1 Laboratory of Biotechnology, Environment, Agri-Food and Health (LBEAS), Faculty of Sciences Dhar El Mahraz, Sidi Mohamed Ben Abdellah University (USMBA), Fez B.P. 1796, Morocco; elouahdanikhadija@gmail.com (K.E.O.); Hamza.mechchate@usmba.ac.ma (H.M.); Amina.bari@usmba.ac.ma (A.B.); dalila.bousta@usmba.ac.ma (D.B.); 2 Biology Department, College of Science, Imam Mohammad Ibn Saud Islamic University (IMSIU), Riyadh 11623, Saudi Arabia; MMyAlzahrani@imamu.edu.sa (M.A.-z.); AAQurtam@imamu.edu.sa (A.A.Q.); msaleissa@imamu.edu.sa (M.A.)

**Keywords:** *Thymus algeriensis*, *Artemisia herba-alba*, antioxidant, anti-inflammatory, analgesic, toxicity

## Abstract

The study of bioactive molecules of natural origin is a focus of current research. *Thymus algeriensis* and *Artemisia herba-alba* are two medicinal plants widely used by the Moroccan population in the traditional treatment of several pathologies linked to inflammation. This study aimed to evaluate the single and combined antioxidant, anti-inflammatory and analgesic effects of the essential oils extracted from these two medicinal plants, and also their potential toxicity. Essential oils were extracted using hydro-distillation in a Clevenger-type apparatus. The antioxidant activity was evaluated by two methods: the scavenging of the free radical DPPH, and the reduction in iron. Anti-inflammatory activity was evaluated by evaluating the edema development induced by carrageenan injecting, while the analgesic power was evaluated according to the number of abdominal contortions induced by the intraperitoneal injection of acetic acid (0.7%). The acute oral toxicity was performed to assess the potential toxicity of the studied EOs, followed by an analysis of the blood biochemical parameters. The results of the two antioxidant tests indicated that our extract mixture exhibits good iron reduction capacity and very interesting DPPH free radical scavenging power, with an IC_50_ of around 4.38 ± 0.98 μg/mL higher than that of the benchmark antioxidant, BHT. The anti-inflammatory test demonstrated that the mixture administered orally at a dose of 150 mg/kg has a better activity, exceeding that of 1% Diclofenac, with a percentage of maximum inhibition of the edema of 89.99 ± 4.08. The number of cramps in the mice treated with the mixture at a dose of 150 mg/kg is significantly lower (29.80 ± 1.92) than those of the group treated with Tramadol (42.00 ± 2.70), respectively. The toxicity results show no signs of toxicity with an LD_50_ greater than 150 mg/Kg. These interesting results show that the two plants’ EOs had an important anti-inflammatory, analgesic, and antioxidant activity, and also a powerful synergistic effect, which encourages further in-depth investigations on their pharmacological proprieties.

## 1. Introduction

In the global approach of the evaluation of the commonly used essential oils by the populations and to study their probable toxicity or undesirable effect, two Moroccan plants were investigated, namely *Artemisia herba-alba* and *Thymus algeriensis.*

From the Lamiaceae family, the genus *Thymus* is among the most widespread genera of medicinal plants in the Mediterranean region with 215 species [1]. In Morocco, there are twenty-one species of *Thymus*, including *T. algeriensis*, *T. ciliatus*, and *T. capitatus*. Notably, this genus is characterized by several pharmacological activities, including anti-inflammatory [2], anti-oxidant [3], antispasmodic [4], and antimicrobial activities [5]. *T. algeriensis* essential oil is well known for its antioxidant, allelopathic, insecticidal, antibacterial, and antifungal proprieties [6,7].

*Artemisia herba-alba* is a species in the Asteraceae family. It is a perennial, silvery-greenish dwarf shrub grows in arid and semi-arid climates in the deserts of Spain, North Africa, and the Middle East, extending to the northwest of the Himalayas [8]. It is widely used in traditional medicine against several pathologies, including diabetes, colds, coughs, intestinal disorders, and in the treatment of human and livestock injuries [9]. The essential oil of this plant is well studied for its antibacterial, antifungal, antiacetylcholinesterase, and also antiproliferative [10,11,12].

This study serves to promote Moroccan medicinal and aromatic plants, and it focuses on two medicinal plants from the region of Imizar-Azilal (Great Atlas of Morocco), which are widely used by the indigenous population against several pathologies in a single and combined mixture, and aimed to evaluate their anti-inflammatory and analgesic potential, along with an assessment of their toxicity with a clear identification of their composition using chromatographic techniques (GC/MS).

## 2. Results

### 2.1. Extraction Yields

The aerial parts of *T. algeriensis* had a relatively low yield rate of ~0.4%. However, the aerial parts of *A. herba-alba* provided a yield of 0.6%.This finding agrees with that of Hazzit et al. [1] for the same species (0.4%); it is similar to that found by Amarti et al. (2011) for the Rchida region in eastern Morocco (0.3%) [5], and it is much lower than that obtained by Dob et al. [13] for the aerial parts of T. algeriensis (1.13%). This yield is higher than that found by Tilaoui et al. [10], who used the aerial parts of *A. herba-alba* from the Imilchil region of Er-rachidia province. Several factors influence the yield of plant essential oils, including the plant species, environmental conditions, harvest time, and extraction technique used [14].

### 2.2. Chromatographic Analysis

The results of chromatographic analysis of the essential oil of *A. herba-alba* revealed the presence of 29 compounds (Table 1), which represent 99.71% of the total EO of the plant, characterized by the presence of chrysanthenone and camphor as main constituents with concentrations respectively of 47.71 and 21.45%, and the absence of caryophyllene. The dominance of chrysanthenone and camphor can be explained by their biosynthesis, which are favored, with the predominance of chrysanthenone from pinenes and their derivatives during the flowering period of plants (March, April, and June).

The chemical composition of the EO of *T. algeriensis* is characterized by the presence of 21 compounds, which represent 90.61% of the totality with a predominance of thymol, borneol, and carvacrol with respectively 46.03, 20, 38, and 5.86% (Table 1). (Chromatograms of both analyses are available in Supplementary Materials). 

### 2.3. Antioxidant Activity of Essential Oils

**Figure 1:**
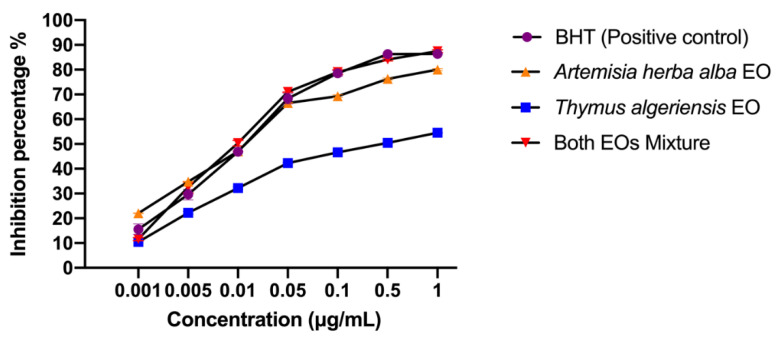
Antioxidant activity profile of butylhydroxytoluene (BHT), *T. algeriensis* and *A. herba-alba* essential oils and their mixture (50:50) in the DPPH test. The values are expressed as the mean ± standard error of the mean with *n* = 3.

From the values obtained, we calculated the percentage of inhibitions by using the formula (in the method below). Based on these percentages of inhibition, it was possible to draw the curves presented in Figure 1, which show variation in the percentage of inhibition as a function of the concentration of essential oil. These curves allowed us to calculate the IC_50_ values presented in Table 2.

Our findings revealed that the mixture of essential oils from the two study plants exhibited the lowest IC_50_ of around 4.38 ± 0.98 μg/mL, which shows substantial inhibition of the oxidation induced by the DPPH radical. This antioxidant activity remains greater than that of BHT, a reference antioxidant, which had an IC_50_ of 6.16 ± 0.28 μg/mL. Additionally, the essential oils of *T. algeriensis* and *A. herba-alba* were less effective at free radical scavenging than the reference substance, with IC_50_ values of 67.85 ± 1.21 μg/mL and 7.84 ± 0.72 μg/mL, respectively.

**Figure 2:**
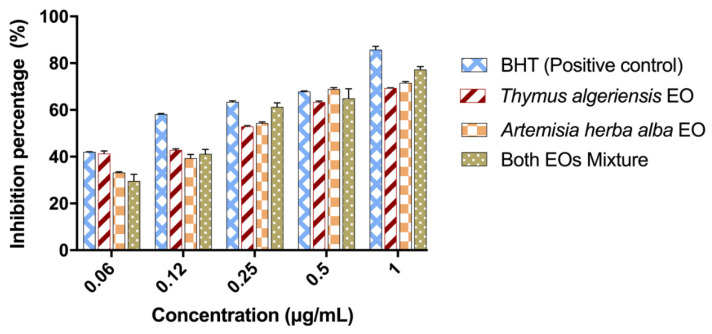
Histogram of the variation in the percentage inhibition as a function of different concentrations in the FRAP assay. The values are expressed as the mean ± standard error of the mean, *p* < 0.05 is considered significant, compared to the control (*n* = 3).

The reduction of ferric iron (Fe^3+^) to ferrous iron (Fe^2^) is one of the most widely used methods to assess the antioxidant potential of compounds in medicinal plants. We used the FRAP method to confirm the antioxidant power of the essential oils and their mixture. Our results allowed us to plot histograms of the inhibition percentage potential of our samples as a function of different concentrations (Figure 2). These results show that the mixture and the separate essential oils had a lower iron reduction capacity than that of BHT and the mixture. Notably, the more the concentration increased, the more the reducing power of our samples increased, which means that the iron reduction capacity is proportional to the increase in concentration.

### 2.4. Anti-Inflammatory Activity of Essential Oils

**Figure 3:**
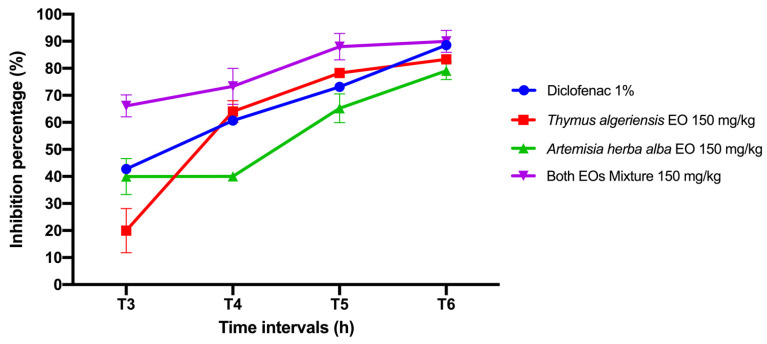
Anti-inflammatory effect of essential oils of *T. algeriensis* and *A. herba-alba* and their mixture administered orally at the dose of 150 mg/kg and Diclofenac 1% on the edema induced by carrageenan in rats. The values are expressed as mean ± standard error of the mean, * *p* < 0.05 compared with control (Diclofenac) (*n* = 5).

The results obtained at the end of this pharmacological test show that the mixture, composed of the two essential oils of *T. algeriensis* and *A. herba-alba*, administered orally at a dose of 150 mg/kg significantly reduced edema, and demonstrated the best edema inhibition potential with a percentage of inhibition of 89.99 ± 4.08, and under the same conditions, Diclofenac 1% had a maximum inhibition of 88.57 ± 0.81 at the sixth hour after the injection of carrageenan. Thus, the essential oils of *T. algeriensis* and *A. herba-alba* at a dose of 150 mg/kg inhibit the edema by 83.33 ± 00 and 79.11 ± 3.22, respectively. This finding suggests that both herbs have a lower anti-inflammatory effect than that of the mixture and Diclofenac 1%. Thus, our samples inhibited edema in a dose-dependent manner and in all phases (Figure 3).

### 2.5. Analgesic Activity

**Figure 4:**
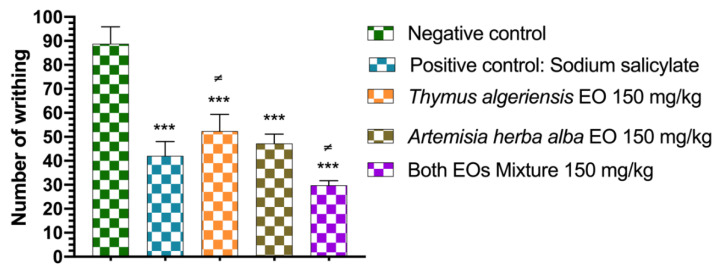
Analgesic activity of the studied essential oils and their mixture. The values are expressed as the mean ± standard error of the mean (*n* = 5), ***: *p* < 0.001 compared to negative control ≠ *p* < 0.05 compared to the positive control.

The results of this test are shown in Figure 4. Notably, the mice treated with the mixture, at a dose of 150 mg/kg, significantly lowered the number of abdominal contractions (29.80 ± 1.92) better than that of the control group (42.00 ± 2.70), which was treated with the reference analgesic drug Tramadol. The essential oils of *T. algeriensis* (52.40 ± 3.10 contractions) and *A. herba-alba* (47.20 ± 1.74) administered at a dose of 150 mg/kg had a slightly lower analgesic effect than that of Tramadol and the mixture.

### 2.6. Acute Toxicity Study

**Figure 5:**
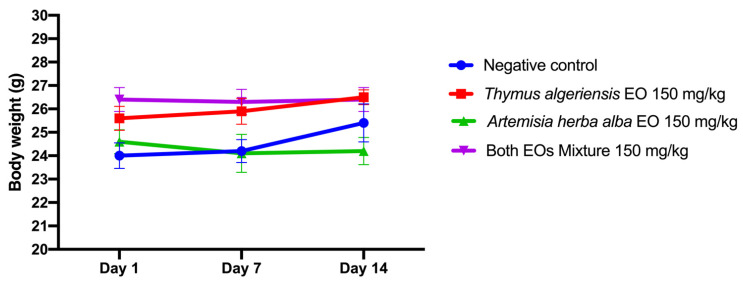
Variations in the weight of the mice as a function of time and the dose of *T. algeriensis* and *A. herba-alba* and their mixture administered orally. Values are presented as mean ± standard error of the mean (*n* = 5).

This toxicity study was adapted from that of OECD guideline 423 [15]. The results of the study on the acute toxicity of the essential oils of *T.* *algeriensis* and *A.* *herba-alba* individually showed that oral administration of single and combined mixture of the two essential oils did not cause any abnormal behavior (sign of toxicity) or death in the mice (Table 3). All animals survived after 14 days of testing. Their body weight remained almost stable over time, and there was no significant difference in the variation in body weight between the treated mice and those of the control for 14 days (Figure 5). These results imply that the LD50 of this mixture is greater than 150 mg/kg, according to the Globally Harmonized System of Classification and Labeling of Chemicals.

The relative organs weight measured at the end of the study and the sacrifice of the mice treated with the single and combined mixture (150 mg/kg) of the essential oils shows no significant difference between the treated and the control in term of liver, kidney, and spleen weight (Table 4). The biochemical analyzes also showed no significant increase in the levels of ASAT and ALAT in all groups (Table 5), compared with the control.

## 3. Discussion

Essential oils are nowadays considered one the main source of bioactive compounds with enormous health benefits [16,17]. Studies of EOs proved their efficacy against several acute and chronic disease such as ROS, inflammatory related diseases, infectious diseases, and many others [18,19]. This study attempted to evaluate the bioactivity of two plants commonly used in the high Atlas of Morocco, namely *T. algeriensis* and *A. herba-alba* in term of antioxidant, analgesic, and anti-inflammatory activities, with an assessment of the plants’ toxicity in single and combined mixture.

The antioxidant activity of a compound reflects its ability to overcome oxidation induced by free radicals [20]. Several methods are currently used to assess this activity. In this study, the free radical scavenging power of the studied essential oils and their mixture was evaluated using two methods: DPPH and FRAP. The EO of *T. algeriensis* IC_50_ value shows low anti-radical power, similar to the chemotype from eastern Morocco characterized by Amarti et al. [5], as a weak antioxidant with an IC_50_ of 745.6 μg/mL. However, the strong antioxidant activity of *A. herba-alba* oil may be due to its major phytochemical compounds chrysanthenone and camphor, which may act as singular oxygen and hydrogen donors [21]. The antioxidant activity of the mixture of the two essential oils gives superior results, compared to the singular effect which indicated a perfect synergy of the EOs leading to a better activity. 

Steroidal anti-inflammatory drugs (NSAIDs) are used to manage several pathologies, including pain, fever, and inflammation [22]. These drugs have several undesirable effects, such as causing kidney disorders and gastrointestinal ulcers, resulting from the inhibition of COX2 (Cyclooxygenase 2), which is a constitutive protein that plays an important role in maintaining tissue integrity [23]. These undesirable effects lead the search for new bioactive compounds from medicinal plants, as an alternative [24,25]. Injection of the carrageenan into the rats’ paws in this study caused the release of pro-inflammatory mediators (chemical mediators that stimulate the inflammatory process), including histamine, serotonin, bradykinin, and prostaglandins [26]. The use of EOs for both plants at the dose of 150 mg/kg demonstrated a powerful anti-inflammatory activity that can be compared to that of Diclofenac, used a positive control in this study. According to Sobeh et al., the alcoholic extract of *T. algeriensis* from Algeria demonstrated its potentiality as a COX-2 inhibitor to be even higher than Celecoxib and Diclofenac, used as positive control [27]. The synergic activity in this anti-inflammatory test was also powerful with a percentage of inhibition of edema higher that all other single treatments.

To assess the analgesic activity of the studied EOs, the writhing test was performed. Following the intraperitoneal injection of acetic acid into rodents, the peripheral nociceptive mechanism was promoted by releasing many chemical mediators, such as histamine, prostaglandins PGE2 and PGEα, serotonin, and bradykinin [28]. In a different study, the methanolic extract of *Vitex congolensis,* which belongs to the same family of *T. algeriensis* (Lamiaceae), shows an excellent analgesic activity at a dose of 300 mg/kg [24]. The mixture of the two essential oils of *T. algeriensis* and *A. herba-alba* at a dose of 150 mg/kg has a highly significant analgesic effect. 

The bioactivity of the EOs and their combination could be attributed to some of the major components identified with the chromatographic analysis, such as thymol.

The study of Marsik, et al. indicates the potential of thymol as an anti-inflammatory molecule at dose of 100 mM via alteration of the prostaglandin biosynthesis by inhibiting cyclooxygenase (COX) [29]. In another in vivo study, thymol isolated from essential oils of *Lippia gracilis* leaves demonstrated a powerful ability to inhibit carrageenan-induced edema formation at the dose of 200 mg/kg [30]; other studies reported the molecule’s ability to inhibit T cell immune response, improve T-helper cells-1 (Th1) [31], and inhibit lipid peroxidation, glycation, dyslipidemia [32]. It was also reported that thymol exhibits its analgesic activity via the nerve cell a2-adrenergic receptors [33].

Beside the obtained results, the toxicity study indicates the safety of the EOs and their mixtures at the studied dose (150 mg/kg).

## 4. Materials and Methods

### 4.1. Plant Material

The plant material used in this study consists of aerial parts (stems, leaves, and flowers) of *T. algeriensis* (BPRN76) and *A. herba-alba* (BPRN16). They were collected during the flowering period (March 2019) in the Imizar-Azilal region (High Atlas of Morocco). The harvested parts were then dried away from sunlight, at atmosphere temperature.

### 4.2. Extraction of Essential Oils

The essential oils were extracted using hydrodistillation in a Clevenger-type apparatus. A measure of 100 g of plant material was distilled with 1 L of water for 2 h in a 2 L flask, surmounted by a 60-cm-long column connected to a condenser. The yields of these distillations were calculated based on the dry weight of plant material dried for 48 h in an oven at 35 °C. The obtained essential oils were stored at a temperature of 4 °C in the presence of anhydrous sodium sulfate in the dark, for the purpose of chemical analysis and biological tests. The average yield of essential oil from the two study plants was calculated based on the plant material of the dry aerial parts.

### 4.3. Chromatographic Analysis

Analyses of the chemical composition of our essential oils were carried out by gas chromatography, coupled with mass spectrometry (GC-MS). The latter was carried out using a gas-phase chromatograph of the Trace GC ULTRA type, equipped with an HP-5 capillary column (5% diphenyl, 95% dimethylpolysiloxane) 30 m in length, 0.25 mm in diameter, and 0.25 μm in film thickness, as well as an injector in split mode 1:10 with a temperature of 250 °C. The detector is of the FID type (temperature of 250 °C). The carrier gas used was helium, with a flow rate of 1.4 mL/min. The column temperature was programmed for 4 °C/min from 50 to 200 °C, and a 5 min dwell at the final temperature. Coupling with the Polaris Q MS mass spectrometer was performed with an interface temperature of 300 °C. The database used was NIST98.

### 4.4. Antioxidant Activity

The antioxidant properties of the essential oils of the two species and the mixture were evaluated using two methods: the first method uses DPPH (2,2-Diphenyl Picrylhydrazyl) as a relatively stable radical molecule [34], and the second method considered the ferric reducing antioxidant power (FRAP), and is based on the reduction of the complex ferric ion and 2,3,5-triphenyl-1,3,4-triaza-2-azoniacyclopenta-1,4-diene chloride (TPTZ) to the ferrous form at low pH [31]. We used the FRAP method to confirm the antioxidant power of the essential oils and their mixture.

#### 4.4.1. DPPH Assay

The DPPH (Sigma-Aldrich, St. Louis, MO, USA) radical scavenging activity of the two essential oils was evaluated following Yeo and Shahidi [34]. The reaction was conducted in a total volume of 2 mL containing 0.4 mL of DPPH (0.004% (*w*/*v*), solubilized in methanol. The essential oils were dissolved in absolute methanol at a concentration of 1.25 μL/mL (stock solution). Then, a series of dilutions were prepared to obtain final concentrations of 0.0005%, 0.001%, 0.005%, 0.01%, 0.05%, and 0.1%. The positive control was prepared with BHT in the same concentrations. The blank was prepared with absolute methanol alone. Tests were repeated three times for each concentration, and the absorbance was performed with a spectrophotometer at 517 nm after incubation for 30 min in the dark.

#### 4.4.2. Ferric Reducing Antioxidant Power (FRAP)

Spectrophotometric analysis of the reducing power of essential oils reducing ferric ion was conducted using the method developed by Benzie Et Strain (1999) [35]. A measure of 3 mL of the prepared FRAP reagent was mixed with 100 µL of the diluted sample, and the absorbance at 593 nm was recorded after a 30 min incubation at 37 °C. FRAP values can be obtained by comparing the change in absorbance in the test mixture with those obtained by increasing the concentrations of Fe^3+^, and they can be expressed in mM of Fe^2+^ equivalents. The essential oils were diluted by adding 1 mL of each sample to different concentrations in absolute methanol, mixed with 2.5 mL of a phosphate buffer solution (0.2 M; pH: 6.6) and 2.5 mL of a solution of potassium ferricyanide K_3_Fe (CN) 6 at 1%. Then, after cooling to room temperature, the tubes were incubated for 20 min at 50°C. Next, 2.5 mL of 10% trichloroacetic acid (TCA) was added to stop the reaction in each tube. The tubes were then centrifuged at 3000 revolutions/min for 10 min. Then, 2.5 mL of absolute methanol and 500 μL of an iron chloride solution (FeCl_3_, 6H_2_O) at 0.1% was added to a sample of 2.5 mL of the supernatant from each tube, and the absorbance of the solution was measured at 700 nm against white in the UV spectrophotometer. BHT was used as a positive control.

### 4.5. Anti-Inflammatory Activity: Carrageenan Edema

The anti-inflammatory activity of our essential oils was evaluated following Winter’s method [36]. In this test, we used rats as animal models, as they are widely used to assess the anti-inflammatory potential of compounds. The rats were randomly divided into four groups of five rats each. The first two groups of rats orally received the essential oils of *T. algeriensis* and *A. herba-alba* at a dose of 150 mg/Kg; the third group of rats received an equal mixture of the two essential oils described above at a dose of 150 mg/Kg, and the last group of rats served as a positive control (received Diclofenac 1%). All groups received their treatments 1 h before the injection of 1% carrageenan (prepared in 0.9% NaCl), which was injected under the plantar fascia of the right hind paw. Measurements of the volumes of the right hind paw of each rat were taken before the induction of edema, and after each hour from the third until the sixth hour after the carrageenan injection. The percentage inhibition of inflammation was calculated using the following formula:

Inhibition = ((St − S0) Control − (St − S0) Treated)/((St − S0) Control) × 100

S0 is the circumference before injection of carrageenan, and St is the circumference at a given time after administration of carrageenan.

### 4.6. Analgesic Activity: Writhing Test

The analgesic potential of the essential oils was evaluated using the number of abdominal contortions induced by the intraperitoneal injection of acetic acid (0.7%). Contractions induced by an intraperitoneal injection of acetic acid are a widely used method to study the peripheral analgesic effect of substances. Groups of five mice were formed. The control group received 0.9% NaCl, whereas the other groups received the essential oils orally at a dose of 150 mg/Kg, the mixture of the EOs at a dose of 150 mg/Kg, and Tramadol. Then, 1.5 h after the administration of the essential oils, the mice received 0.7% acetic acid intraperitoneally at a dose of 10 mL/kg. After a lag time of 5 min after the acetic acid injection, the number of contortions was counted for each group over the next 30 min [37].

### 4.7. Acute Oral Toxicity

The acute oral toxicity was tested using an approach adapted from Test Guideline 423 (OECD, 2001) [15,38]. Acute gavage was performed using an intragastric tube in two-month-old mice. The mice were divided into five groups of five mice each. The groups were treated orally with the single and combined mixture of both essential oils (*T. algeriensis* and *A. herba-alba*) at a dose of 150 mg/kg. The behavior of the mice was observed daily, and their weights were measured daily. Observations focused on general appearance, mobility, sensitivity to noise, diet, the appearance of feces and mortality. After 14 days, the treatment ended, and the animals were sacrificed. A blood sample was obtained for each mouse using a heparinized tube to assay the biochemical parameters: AST, ALT, creatinine, and urea. Then, the liver, kidneys, and spleen were removed, and weighed to assess the relative organ weight.

### 4.8. Biochemical Parameters

The tubes containing the blood samples were centrifuged at 2500 rpm for 15 min. Then, the serum was collected to assay the four biochemical parameters, creatinine, and urea and hepatic enzymes ASAT and ALAT. The hepatic enzymes were assayed using the colorimetric method of Reitman and Frankel [39], creatinine was assayed using the picrate reaction method in an alkaline medium, and the urea content was measured using the colorimetric method in a diacetyl monoxime.

### 4.9. Statistical Analysis

Statistical analyses were performed using Graph Prism software version 8.0.1. for Windows, and one-way analysis of variance was used to examine differences between the means of treatment groups and the control, and the differences were considered significant if *p* < 0.05.

## 5. Conclusions

The significant results obtained during this pharmacological study have shown that the single and the mixture composed of essential oils of *T. algeriensis* and *A. herba-alba* contain bioactive compounds endowed with a strong antioxidant, anti-inflammatory, and analgesic activity compared to the reference drug. These results constitute a scientific basis that justifies the frequent traditional use of *T. algeriensis* and *A. herba-alba* in the management of several pathologies related to pain and inflammation. This open new possibilities of use of those essential oils to more precise inflammatory diseases or other applications related to the food or pharmaceutical industry.

## Figures and Tables

**Table 1:** Chemical compounds of *A. herba-alba* and *T. algeriensis* essentials oils.

N	K	Compound	*T.a* %	*A.h* %	N°	IK	Compound	*T.a* %	*A.h* %
**1**	926	Tricyclene	**-**	**0.25**	**25**	1165	Borneol	**20.38**	**-**
**2**	931	α-Thujene	**0.64**	**0.54**	**26**	1177	Terpin-4-ol	**0.48**	**0.39**
**3**	939	**α-Pinene**	**0.35**	**4.31**	**27**	1189	α-Terpineol	-	**0.44**
**4**	948	Camphene	**-**	**0.38**	**28**	1204	Verbenone	**0.08**	**-**
**5**	973	Sabinene	**0.56**	**-**	**29**	1205	Piperitol, trans-	**-**	**0.40**
**6**	980	β-Pinene	**-**	**1.07**	**30**	1235	Thymol methyl ether	**0.98**	**-**
**7**	991	β-Myrcene	**-**	**0.24**	**31**	1258	Myrtanol, trans-	**-**	**0.56**
**8**	1011	δ-3-Carene	**3.1**	**-**	**32**	1271	Neo-3- thujyl acetate	**-**	**0.57**
**9**	1018	α- Terpinene	**2.3**	**0.20**	**33**	1282	α-Terpin-7-al	**0.18**	**0.46**
**10**	1026	p-Cymene	**0.47**	**-**	**34**	1290	**Thymol**	**46.03**	**-**
**11**	1030	Limonene	**-**	**1.14**	**35**	1291	3-thujyl acetate	**-**	**-**
**12**	1031	1,8-Cineole	**2.63**	**-**	**36**	1298	**Carvacrol**	**5.86**	**-**
**13**	1048	β-Ocimene,(E)-	**2.80**	**-**	**37**	1315	Neo-iso-acetate isopulegol	**-**	**0.61**
**14**	1067	Sabinene-hydrate, cis-	**0.54**	**-**	**38**	1339	dihydro-α terpinyl- acetate trans-	**-**	**0.42**
**15**	1068	γ-Terpinene	**0.74**	**0.19**	**39**	1373	β-elemene	**-**	**2.81**
**16**	1102	**α-thujone**	**-**	**4.02**	**40**	1480	Germacrene D	**-**	**3.15**
**17**	1114	β-thujone	**-**	**0.57**	**41**	1499	α-Muurolene	**-**	**1.14**
**18**	1123	**Chrysanthenone**	**-**	**47.71**	**42**	1568	Caryophyllene alcohol	**-**	**0.25**
**19**	1138	α-Campholenal	**-**	**0.80**	**43**	1574	Germacrene D-4-ol	**-**	**0.37**
**20**	1143	β-dihydroterpineol, cis-	**-**	**21.59**	**44**	1581	Caryophyllene oxide	**0.28**	**-**
**21**	1159	**Camphor**	**-**	**1.81**	**45**	1586	Davanone	**-**	**1.13**
**23**		β-dihydroterpineol, trans-	**1.87**	**-**	**46**	1700	Caryophyllene acetate	**0.34**	**-**
**24**	1163	Isoborneol	**-**	**2.19**					
**Total**								**90.61**	**99.71**

**Table 2:** The concentrations of the essential oils of *Thymus algeriensis*, *Artemisia herba-alba*, their mixture (50:50), and butylhydroxytoluene (BHT) that inhibit 50% of antioxidant activity (IC_50_) in the DPPH test.

Sample	IC_50_ (%)
*Thymus algeriensis*	67.85 ± 1.21
*Artemisia herba-alba*	7.84 ± 0.72
Mixture	4.38 ± 0.98
BHT	6.16 ± 0.28

Values are expressed as the mean ± standard deviation (*n* = 3).

**Table 3:** Results of the observation of the mice every day for 14 days after oral administration of *T. algeriensis* and *A. herba-alba* and their mixture.

Clinical Signs of Toxicity/Lot	Group 1 (Negative Control)	Group 2 *T. algeriensis* (150 mg/kg)	Group 3 *A. herba-alba* (150 mg/kg)	Group 4 Mix (150 mg/kg)
Drowsiness	−	−	−	−
Anorexia	−	−	−	−
Diarrhea	−	−	−	−
Breathing difficulties	−	−	−	−
Sensitivity to pain and noise	−	−	−	−
Abdominal pain (contortion)	−	−	−	−
Convulsion	−	−	−	−
Tremor	−	−	−	−
Coma	−	−	−	−
Mortality	−	−	−	−

**Table 4:** Effect of the essential oil mixture administered orally on the weight of organs removed from mice after 14 days of treatment.

	Negative Control	*T. algeriensis* 150 mg/kg	*A. herba-alba* 150 mg/kg	Mix 150 mg/kg
Liver	1.507 ± 0.104	1.533 ± 0.206	1.591 ± 0.285	1.540 ± 0.189
Kidney	0.450± 0.005	0.463 ± 0.07	0.480 ± 0.130	0.436 ± 0.06
Spleen	0.154 ± 0.006	0.161 ± 0.052	0.164 ± 0.071	0.151 ± 0.096

Values are presented as mean ± standard error of the mean (*n* = 5).

**Table 5:** Values of biochemical parameters of control and treated mice after 14 days after the single administration of essential oils of *T. algeriensis*, *A. herba-alba*, and the mixture at a dose of 150 mg/kg.

Parameters	Negative Control	*T. algeriensis* 150 mg/Kg	*A. herba-alba* 150 mg/Kg	Mix 150 mg/Kg
“Urea (g/L)”	0.39 ± 0.03	0.33 ± 0.04	0.41 ± 0.03	0.37 ± 0.03
“Creatinine (mg/L)”	3.67 ± 0.33	3.83 ± 0.16	3.59 ± 0.33	4.05 ± 0.57
“ASAT (UI/I)”	196 ± 4.72	202 ± 8.03	222.33 ± 5.54	181.33 ± 8.36
“ALAT (UI/I)”	26.67 ± 3.84	21 ± 3.05	22.33 ± 3.38	25.8 ± 1.26

Values are presented as mean ± standard error of the mean (*n* = 5).

## Data Availability

Data are available upon reasonable request.

## Supplementary Materials

The following are available online, Figure S1: Chromatogram of *Artemisia herba* *alba* essential oil; Figure S2: Chromatogram of *Thymus algeriensis* essential oil.

## Sample Availability

Samples of *Thymus algeriensis* and *Artemisia herba-alba* are available from the authors.
