# Supplementary material for: Thymus algeriensis and Artemisia herba-alba Essential Oils: Chemical Analysis, Antioxidant Potential and In Vivo Anti-Inflammatory, Analgesic Activities, and Acute Toxicity
Source: Molecules. 2021 Nov 10;26(22):6780. doi: 10.3390/molecules26226780 (PMC8625911; doi:10.3390/molecules26226780)
Supplement: Supplementary file 1 [file molecules-26-06780-s001.zip › molecules-1410243-supplementary.pdf]

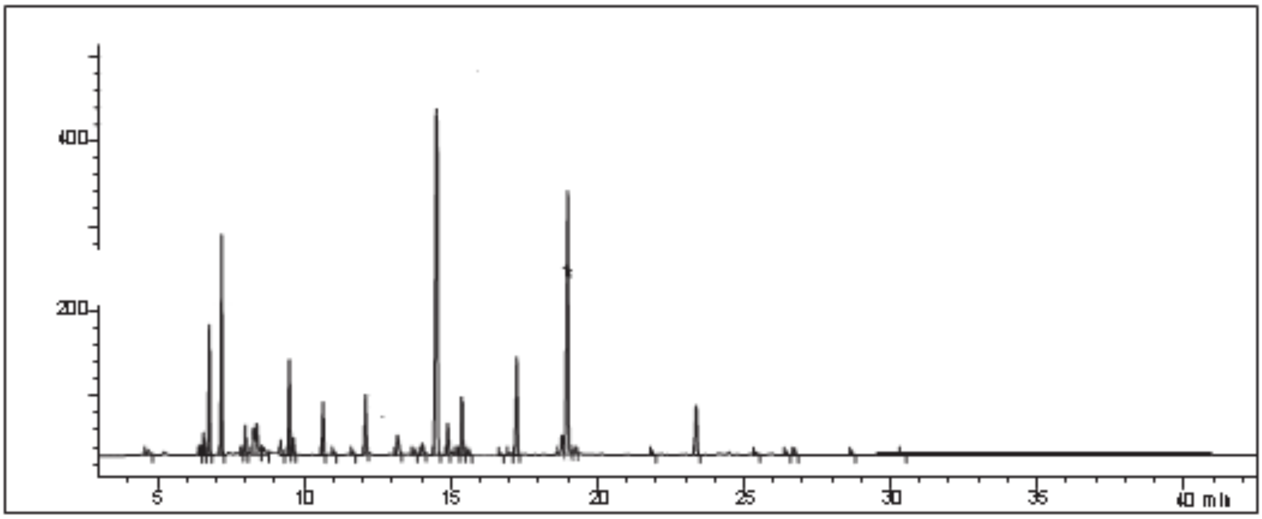

Figure S1. Chromatogram of *Artemisia herba-alba* essential oil

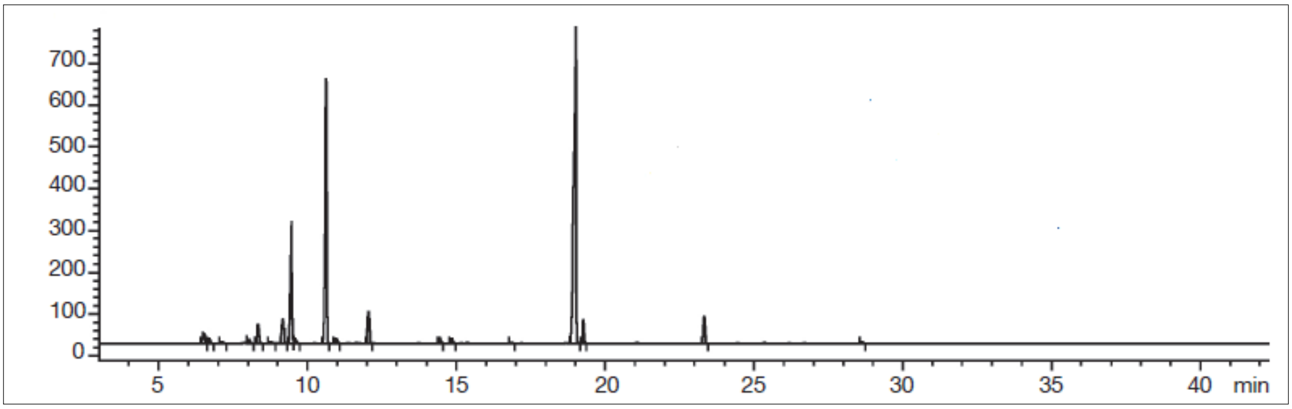

Figure S2. Chromatogram of *Thymus algeriensis* essential oil
